# Case Report: Pediatric age onset CNTN1 antibody-associated neuropathy with nephropathy and literature review

**DOI:** 10.3389/fimmu.2025.1549363

**Published:** 2025-06-18

**Authors:** Ceyda Bayraktar Eltutan, Simon Rinaldi, Atay Vural, Bagdagul Aksu, Hülya Maras Genc, Edibe Pembegul Yildiz

**Affiliations:** ^1^ Division of Pediatric Neurology, Department of Pediatrics, Istanbul Faculty of Medicine, Istanbul University, Istanbul, Türkiye; ^2^ Nuffield Department of Clinical Neurosciences, John Radcliffe Hospital, University of Oxford, Oxford, United Kingdom; ^3^ Research Center for Translational Medicine, Koc University, Istanbul, Türkiye; ^4^ Division of Pediatric Nephrology, Department of Pediatrics, Istanbul Faculty of Medicine, Istanbul University, Istanbul, Türkiye

**Keywords:** chronic inflammatory demyelinating polyradiculopathy, membranous glomerulonephritis, autoimmune nodopathy, anti-contactin antibody, child

## Abstract

We present a 12-year-old boy with acute onset sensorimotor neuropathy and membranous glomerulonephritis associated with contactin-1 antibodies. This prompted us to explore the clinical characteristics of this condition and assess whether its presentation differs between pediatric and adult patients. A comprehensive search was conducted across multiple online databases, including PubMed and EMBASE, using MeSH terms such as “chronic inflammatory demyelinating polyradiculopathy”, “acute inflammatory demyelinating polyradiculopathy “, “CIDP”, “Guillain Barre syndrome”, “proteinuria”, “nephrotic syndrome”, “nephropathy”, “renal disease”, “glomerulonephritis”, “membranous nephropathy”, “autoimmune nodopathies”, and “membranous glomerulonephritis”. We reviewed publications up to October 2024 and identified 39 patients with anti-contactin associated CIDP (chronic inflammatory demyelinating polyradiculopathy) with membranous glomerulonephritis (MGN), including our case. This rare coexistence typically occurs at advanced ages, with only two pediatric cases. Clinical features were similar regardless of age at onset. We compared the onset, symptoms, progression, renal histopathology, and treatment responses between pediatric and adult patients.

## Introduction

Antibodies against neurofascin-155 (Nf155), neurofascin 140/186 (Nf140/186), contactin-1 (CNTN1) and contactin associated protein-1 (Caspr1) are found in some patients with acquired, immune mediated neuropathies (1). In the latest guideline published in 2021 by the Joint Task Force of the European Academy of Neurology and the Peripheral Nerve Society, patients with these autoantibodies are classified as having autoimmune nodopathies, rather than chronic inflammatory demyelinating polyneuropathy (CIDP) (2).

Both acute inflammatory demyelinating polyneuropathies (AIDP) and CIDP are acquired, immune-mediated, peripheral neuropathies. AIDP, an electrophysiologically defined subtype of Guillain Barre Syndrome (GBS), has an acute onset and is usually self-limited, while CIDP has a persistent or relapsing course of disease. The association between acute polyneuropathy and nephropathy has been long reported (3–5). GBS-associated nephropathy usually does not require histological diagnosis, as it often resolves spontaneously. In cases associated with CIDP, the renal disease appears to persist (6). Several reports have described an association between CIDP and nephropathy (7–14). More specifically, the relationship between autoimmune nodopathy and membranous glomerulonephritis (MGN) has recently become a subject of further research and its physiopathology has been partially elucidated (10–12).

MGN is also an immune-mediated disease. Although it is one of the most common causes of nephrotic syndrome in adults, it is rare among children. When MGN develops secondary to an underlying condition -such as infection, malignancy, or autoimmune disease- it is classified as secondary MGN. Primary and secondary MGN differ in their pathological appearances. While both types can show lesions in the glomerular basement membrane, primary MGN typically involves only the capillary walls. In contrast, secondary MGN may present with additional findings such as mesangial hypercellularity, extraglomerular deposits, and full-house immunostaining. While IgG4-predominant staining is associated with primary MGN, IgG1 and IgG2 predominance is expected in secondary MGN (15). In 80% of cases of primary MGN, anti-phospholipase A2 receptor (PLA2R) is positive, while it is mostly negative in secondary MGN. In 15% of cases of primary MGN, the target antigen remains unknown. Previous case reports have suggested an association between antibody mediated neuropathies and nephropathy, indicating that an immune-mediated neuro-renal disease may be targeting both myelin and podocytes (12).

## Case report

A 12-year-old Turkish boy presented with mild, gradually progressive muscle weakness over several weeks. He was unable to walk without support for the last three days. There was weakness of hip flexors, hip extensors, knee flexors, knee extensors, ankle flexors and extensors. Deep tendon reflexes were decreased in the upper extremities and absent in the lower extremities. Vibration sensation was reduced up to the knees. Romberg’s test was positive, suggesting sensory ataxia. Cranial nerve examination was normal. There was no respiratory compromise. The remainder of the examination was normal.

**Figure 1:**
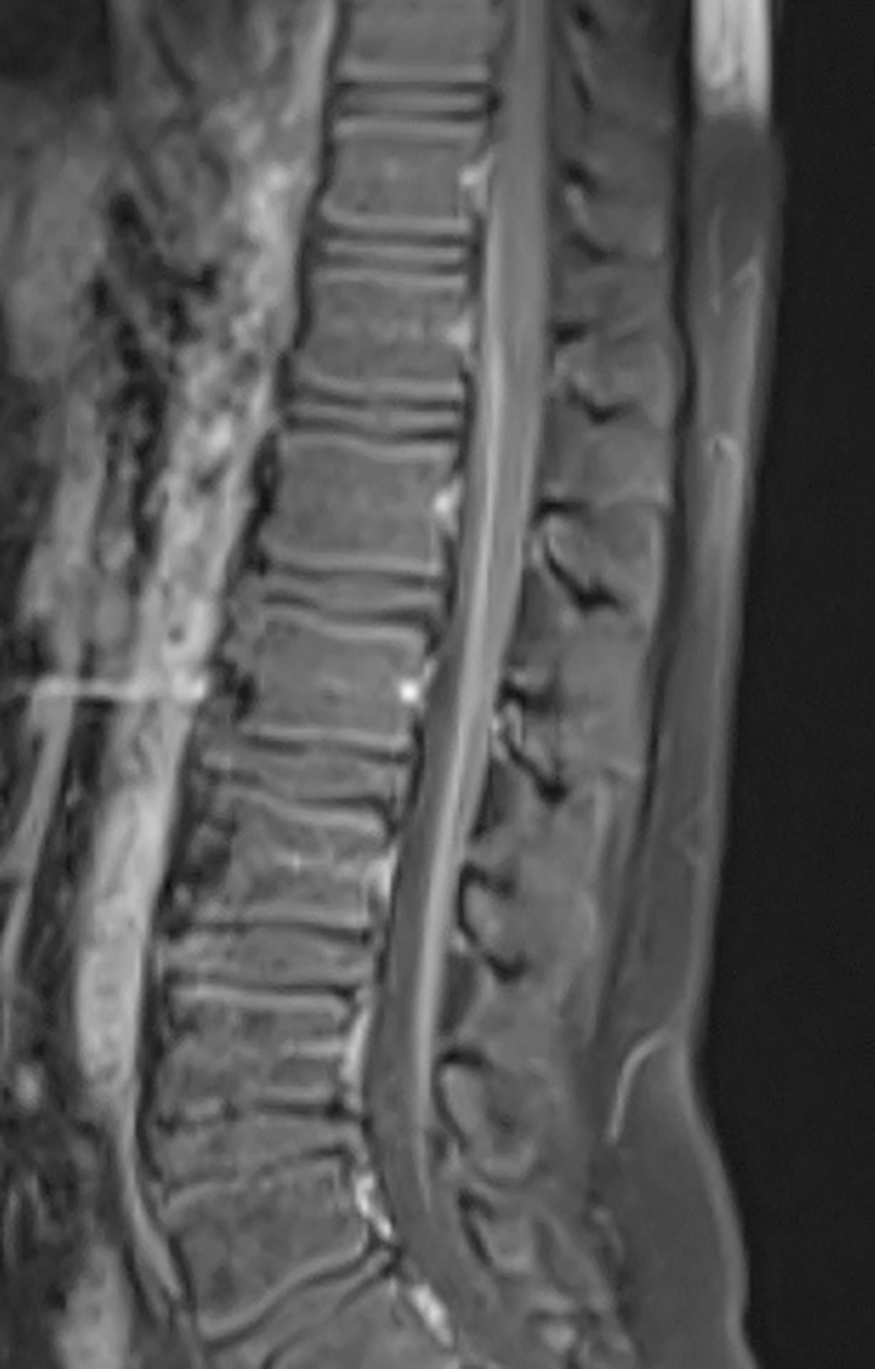
Sagittal T1-weighted post-contrast MRI of the spine demonstrates enhancement of the cauda equina nerve roots.

Cerebrospinal fluid (CSF) analysis revealed elevated protein levels (236 mg/dl, normal value <45 mg/dl) with no leukocytes. Contrast-enhanced spinal cord MRI showed thickening of the spinal roots of the cauda equine with contrast enhancement (**Figure 1**). Nerve conduction studies (NCS) showed significant reduced motor conduction velocity, decreased compound muscle action potentials (CMAPs), and prolonged motor distal latency in bilateral common peroneal and tibial nerves. Sensory nerve action potentials (SNAPs) were absent in the lower extremities and was relatively normal in the upper extremities. Initially he was diagnosed with AIDP and received intravenous immunoglobulin (0,4 gr/kg/d for five days). After the initial treatment there was minimal improvement in lower extremity numbness only. At this time, the modified Rankin Scale (mRS) score was 4.

The patient also had concomitant new-onset nephrotic range proteinuria. Blood urea nitrogen and serum creatinine levels were normal, and there was no edema. A 24-hour urine collection demonstrated 4.2 g/L of protein. Renal ultrasonography was unremarkable. A renal biopsy was performed which was compatible with stage II membranous nephropathy. Light microscopy revealed global sclerosis in 1 out of 20 glomeruli. Direct immunofluorescence showed diffuse granular subepithelial IgG deposits, staining along capillary loops [(IgG (3+), IgM (-), C3 (2+), fibrinogen (-), C1q (-), C4d (+), kappa (2+), lambda (3+)]. PLA2R was negative by immunohistochemistry. There was no amyloid deposition. Electron microscopy showed uniform diffuse thickening of the glomerular basement membrane with the formation of segmental spikes. There was no increase in mesangial cellularity, and the tubules and interstisium showed no significant abnormalities. The absence of mesangial hypercellularity, extraglomerular deposits, and full-house immunostaining, along with subepithelial IgG deposits and spike formation on electron microscopy, were suggestive of primary MGN despite negative PLA2R staining.

Serological tests for possible etiological factors, including hepatitis B virus DNA, antibodies to hepatitis C virus and cytomegalovirus, HIV, and Treponema pallidum, were all within normal limits. There was no significant family history of neurological or renal disease. Complement and immunoglobulin levels were within normal ranges. Serum anti-ganglioside antibodies were negative. Folate, vitamin B12 and HbA1c levels were also within normal ranges. Anti-nuclear antibody was positive with a titer of 1:100. Anti-SSa and anti-Mi antibodies were also positive. Anti-neutrophil cytoplasmic antibodies (ANCA), anti-PLA2R, anti-GBM, and anti-double-stranded DNA (anti-dsDNA) antibodies were all negative. Ophthalmological examination was unremarkabele, and the Schirmer test was negative. The salivary gland biopsy showed sparse periductal lymphocytic infiltration, with no significant inflammatory changes.

**Figure 2:**
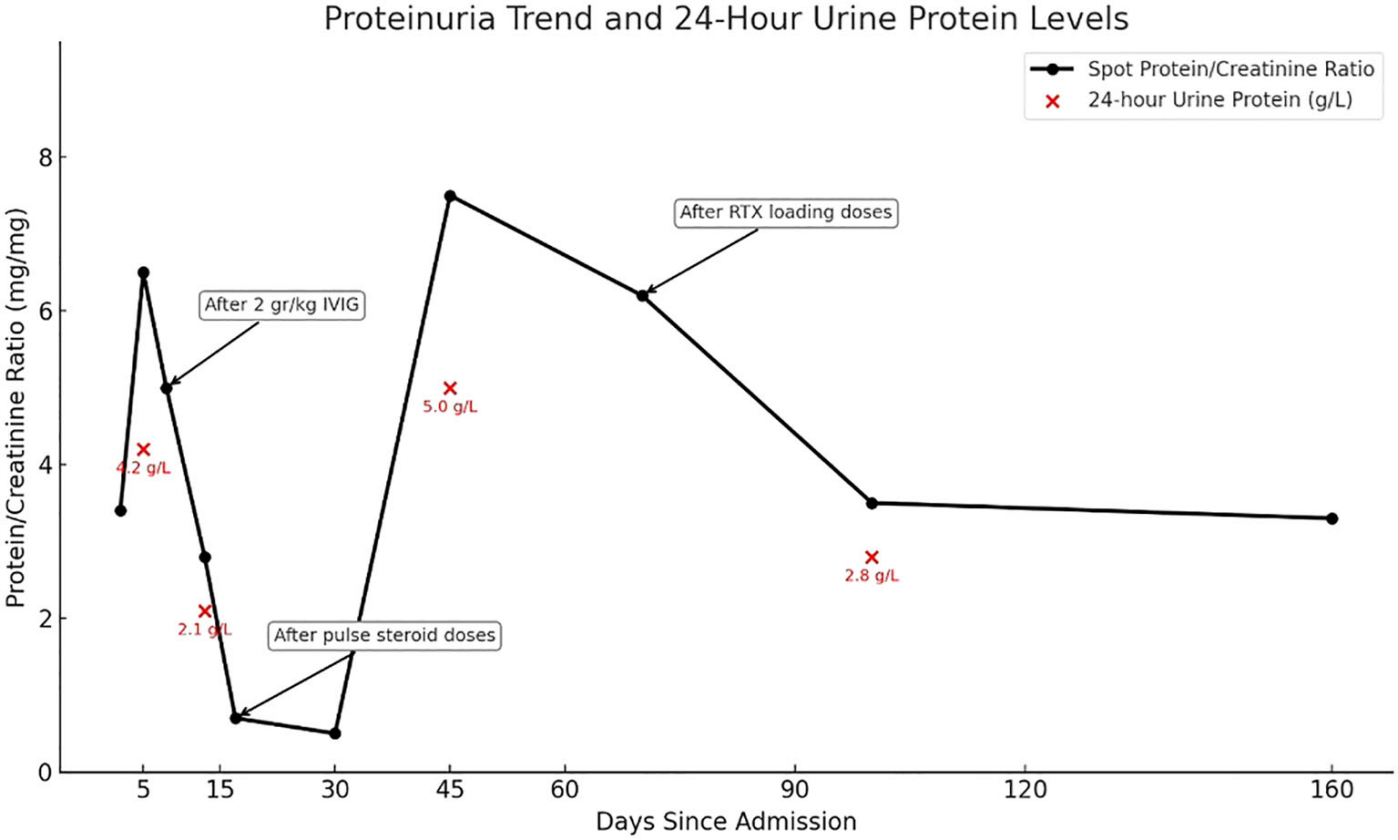
Spot urine protein/creatinine ratio and 24-hour urine protein levels during follow-up. The black line shows spot urine protein/creatinine ratio trends, and red dots indicate 24-hour urine protein levels. Arrows mark the timing og IVIG, pulse steroids, and rituximab treatments.

Due to the progression period exceeding 8 weeks, acute-onset CIDP was diagnosed according to the European Academy of Neurology and the Peripheral Nerve Society (EAN/PNS) (2). We administered intravenous methylprednisolone 1 gr/day for five days. He was able to take a few steps without help, by the fifth day. Neurological symptoms, including ataxia, limb numbness and gait disturbance were slightly alleviated, the mRS score was 3 (**Table 1**). There was only a slight decrease in proteinuria. Due to partial improvement in neurological findings, and incomplete response for nephrologic findings, rituximab therapy was initiated. After 4 weeks of rituximab treatment, neurological symptoms further improved, the mRS score decreased to 2, and the spot urine protein/creatinine ratio gradually declined from about 3–4 to near 1 (**Figure 2**). As autoimmune nodopathy panel testing was not available at our center, the serum sample was sent to the Oxford Autoimmune Neurology Diagnostic Laboratory. Serum samples were analyzed using a live cell-based assay, with initial screening dilutions of 1:100 for NF155 and NF186, and 1:40 for CNTN1/Caspr1. Our patient tested positive for anti-CNTN1 antibodies at screening level, and subsequent subclass analysis revealed IgG1 predominance. Monthly maintenance IVIG therapy was continued. An increase in proteinuria was observed 3–4 months after the loading rituximab doses. He receives regular physiotherapy. On his last examination, five months after rituximab, he exhibited no motor weakness; however, proteinuria persisted at a non-nephrotic level. We follow the patient in terms of relapse of both neurological and renal symptoms.

**Table 1:** Changes in disability scores over time.

Time Point	mRS	INCAT	ONLS Arm	ONLS Leg
On admission	4	5	2	5
After IVIG (2 g/kg)	4	5	2	5
After pulse methylprednisolone	3	3	0	3
After rituximab loading	2	1	0	1
3 months after rituximab	1	0	0	1

## Discussion

We conducted a comprehensive literature review of cases related to our patient, carefully examining the references of relevant articles to avoid any reported patients. To our knowledge, the first pediatric patient of CIDP with MGN was reported in 1992 by Kohli et al. (9), followed by a second case reported by Kanemoto in 1999 (16). Tang et al. later identified the first adolescent-onset case presenting with a GBS-like illness and positive for anti-CNTN1 antibodies (7). As the first two pediatric cases were reported in the 1990s, the clinical information was limited, and anti-contactin antibodies were not studied at that time. Including our case, a total of 39 patients with anti-contactin antibody-associated CIDP and membranous glomerulonephritis (MGN) have been reported in the literature (**Table 2**), with only one previous pediatric case - a 14-year-old-boy- described by Tang et al. (7). Therefore, our patient represents the second pediatric case with this association. This condition predominantly occurs in older adults. Among the 37 adult patients, age at onset ranged from 39 to 80 years, with a mean of 59.7 years. Of these, 25 were male, 6 were female, and gender was not reported in 6 cases.

**Table 2:** Patients with neuropathy and membranous glomerulonephritis with positive anti-CNTN1 antibody.

Patient no	Author	Year	Country	Age	G	Onset course/Time	Anti-CNTN1 ab predominant subtype	CSF protein	Other antibodies
1	**Present case**	**2024**	**Turkey**	**12**	**M**	**Acute / Concurrent**	IgG1	**236**	Anti SSa/Ro, Anti Mi, ANA+
2	**Tang** (7)	**2023**	**China**	**14**	**M**	**Acute / Concurrent**		**284**	
3	Liu (17)	2024	China	65	M	Acute	IgG4	147	
4	Shida (18)	2024	Japan	50	M	Chronic	IgG4	ND	
5-16.	Fehmi (12 patients) (19)	2023	UK	39-79	3F / 9M	Subacute	IgG4	32	
17.	Tang (7)	2023	China	60	M	Chronic		ND	
18.	Tang (7)	2023	China	46	F	Chronic		196	
19.	Santoro (20)	2022	Italy	73	F	Subacute	IgG4	ND	
20.	Remiche (21)	2022	Belgium	65	M	Acute	IgG4	462	
21.	Tan (22)	2022	Malaysia	37	M	Subacute	IgG4	220	
22.	Xu (10)	2021	China	57	M	Subacute	IgG1+, IgG3+, IgG2-, IgG4- (IgG4 predom. in chronic phase)	165	Anti PLA2R+
23.	Plaisier (23)	2021	France	61	M	Subacute	IgG4	79	
24-28.	Le Quintrec (12)	2021	France	58-80	5M	ND	IgG4	ND	
29-34.	Delmont (24)	2020	France	55-75	ND	ND	IgG4	ND	
35.	Nazarali (8)	2020	Canada	43	M	Subacute	IgG4	400	
36.	Cortese (1)	2020	Italy	58	M	Subacute	IgG3, IgG4	ND	
37.	Taieb (25)	2019	France	75	M	Subacute	IgG4	150	
38.	Hashimoto (11)	2018	Japan	78	F	Subacute	IgG4 and IgG1 were dominant)	61	Anti SSa/Ro (1:16)
39.	Doppler (26)	2015	Germany	48	M	Acute	IgG4>IgG3>IgG2>IgG1	204	

Bold values indicate pediatric cases; all others are adult patients.

Previous studies have indicated that anti-CNTN1 associated nodopathy exhibit distinct characteristics (27), which are even more pronounced in patients with concomitant membranous glomerulonephritis (MGN) (10, 11, 14). Xu et al. compared anti-CNTN1 positive patients with and without MGN, finding that those with MGN had an earlier age of onset (10). We observed that the disease onset was more often subacute or chronic in adults. In contrast, both pediatric patients were initially diagnosed with AIDP, which was later revised to CIDP as the disease course progressed. Following the detection of the anti-contactin antibodies, the diagnosis of autoimmune nodopathy was confirmed.

Clinical characteristics of anti-CNTN1 associated nodopathy include acute or subacute onset, rapid progression, worse disability, distal-dominant sensorimotor neuropathy, frequent proprioceptive sensory loss leading to sensory ataxia, elevated CSF protein levels, early axonal involvement, and poor response to intravenous immunoglobulin. The majority of patients including the pediatric cases exhibited these findings. All patients had nephrotic range proteinuria. Renal biopsy showed granular deposits of IgG along the glomerular basement membrane consistent with membranous glomerulonephritis for all patients. High intensity of IgG staining on glomeruli, paucity of C3 deposits and the absence of C1q staining were observed, consistent with primary MGN. Antibodies against phospholipase A2 receptor (PLA2R) were negative in all cases except one. Interestingly, this patient was also one of the three patients who had anti-contactin antibodies with IgG1 subclass predominance (10). Including the other reported pediatric patient, the majority of patients exhibited IgG4 subclass anti-CNTN1 antibodies. IgG1 subclass predominance in three patients was attributed to early sample collection during the acute phase of the disease (26, 28). In the case reported by Xu et al., when retested in the chronic phase, a subclass switch from IgG1 to IgG4 was revealed (10). Notably, all three patients with IgG1 predominance also exhibited positivity for other autoantibodies. In the case reported by Xu et al. anti PLA2R antibodies were positive, and in the case reported by Hashimoto et al. anti SSA/Ro positivity was observed (11). Our patient is the third case with IgG1-predominant anti-CNTN1 antibodies. Serum sample was also obtained during the acute phase, and the additional antibodies -including anti SSA/Ro, anti-Mi and ANA- were also detected.

There are only four reported cases in the literature, in which patients diagnosed with both CIDP and MGN tested negative for anti-CNTN1 antibodies. Two cases were reported by Tang et al. (7), one by Zhang et al. (29), and one by Gupta et al. (30). In the case reported by Zhang et al., renal histopathology was consistent with secondary MGN. In the case reported by Gupta et al., the patient tested positive for PLA2R and exhibited features consistent with Sjogren's syndrome. These findings suggest that the MGN in both patients may be related to a condition other than anti-contactin associated nodopathy. However, in the study by Tang et al., despite the clinical and laboratory findings being highly consistent with autoimmune nodopathy, both patients were tested negative. This raises the question of whether serum antibody testing was performed after immunotherapy, or whether seropositivity might have detected during subsequent follow-up. Several cases were reported prior to 2015, with the coexistence of CIDP with MGN, when anti contactin antibodies were not tested. Given the high probability of antibody positivity in these patients, testing even during follow-up may offer valuable guidance for immunotherapy decisions.

Our literature review revealed that, among patients with contactin-positive nodopathy and nephropathy, membranous glomerulonephritis (MGN) was the only renal pathology reported. Similarly, in patients with anti-neurofascin155 associated nodopathy and nephropathy, the histopathological diagnosis has consistently been focal segmental glomerulosclerosis (FSGS) (31–33). This suggests a potential relationship between the type of autoantibody and the glomerular pathology. The absence of glomerulonephritis in seronegative patients across various cohort studies further supports the hypothesis of an immune-mediated neuro-renal disease (24).

Smyth et al. reported a patient who developed MGN two decades after a CIDP diagnosis, and had been followed for essential hypertension for 10 years prior (34). Therefore, in patients diagnosed with CIDP at a relatively young age, it would be beneficial to follow blood pressure and proteinuria.

Several reports have emphasized that patients with autoimmune nodopathies have poor response to first-line treatments like corticosteroids or IVIG, in contrast to CIDP patients, which show a good response. When we reviewed the overall treatment responses, we found that while IVIG initially improved sensorimotor functions, it was not consistently effective in follow-up, and had minimal impact on proteinuria. The limited response to IVIG treatment in nodopathy is often attributed to the predominance of IgG4 subclass antibodies. Recent studies suggested that IgG1 and IgG3 subclasses may contribute to acute inflammation through complement activation, which could explain the transient initial response to IVIG (11, 35). It is also stated that, a subclass switch to IgG4 during the chronic stage, may explain the subsequent decline in IVIG responsiveness (36, 37). Early identification of autoimmune nodopathies is crucial for guiding treatment and preventing long term axonal degeneration. Corticosteroids provided transient improvement in nephrotic symptoms. Rituximab was generally more effective than both IVIG and corticosteroids in managing both nodopathy and nephropathy (23). However, resistance to rituximab has also been reported (25). In both pediatric cases, the treatment responses were consistent with these overall findings. Furthermore, longitudinal studies are needed to fully understand the complex interplay between autoantibodies and complement activation in autoimmune nodopathies.

## Conclusion

Our case provides further evidence for the association between autoimmune nodopathy and nephropathy in pediatric population. Due to the limited number of reported pediatric cases and their short follow-up durations, it remains unclear whether early-onset correlates with disease severity. The distinct phenotype of anti-contactin-1 mediated nodopathies suggests a shared immune-mediated pathogenesis affecting both the peripheral nervous system and the kidneys. Antibody testing should be considered in male patients with acute or subacute onset of severe sensorimotor neuropathy, especially when response to IVIG and corticosteroids is poor. Although the coexistence of nodopathy and nephropathy is rare, urine analysis for proteinuria and blood pressure measurements may help detect subclinical renal involvement at an earlier stage. Different types of nephrotic syndrome may occur depending on the type of antibody present. Rituximab appears to be an effective treatment option for both neurological and renal involvement in these cases. The main limitation was the inability to repeat antibody subclass testing during follow-up, which could have provided further insight.

## Data Availability

The original contributions presented in the study are included in the article/supplementary material. Further inquiries can be directed to the corresponding author.
